# Screening for Mental Health Problems in US Public Schools

**DOI:** 10.1001/jamanetworkopen.2025.21896

**Published:** 2025-07-18

**Authors:** Jonathan Cantor, Ryan K. McBain, Jacquelin Rankine, Aaron Kofner, Fang Zhang, Alyssa Burnett, Joshua Breslau, Ateev Mehrotra, Bradley D. Stein, Hao Yu

**Affiliations:** 1 RAND, Santa Monica, California; 2 RAND, Arlington, Virginia; 3 Brigham & Women’s Hospital, Boston, Massachusetts; 4 University of Pittsburgh School of Medicine, Pittsburgh, Pennsylvania; 5 Harvard Medical School, Boston, Massachusetts; 6 Harvard Pilgrim Health Care Institute, Boston, Massachusetts; 7 RAND, Pittsburgh, Pennsylvania; 8 Brown University School of Public Health, Providence, Rhode Island

## Abstract

This cross-sectional study estimates the frequency of mental health screening in US public schools and perceptions of school leaders on the ease of connecting youth to mental health care.

## Introduction

In 2021, the US Surgeon General declared a youth mental health crisis.^1^ Schools are strategic locations for screening, treatment, and referral for mental health services that may enhance access for youth with barriers to care in other settings.^2^ In 2016, 13% of school districts reported screening for mental health.^3^ Given the evolving youth mental health crisis after the COVID-19 pandemic, we estimated the frequency of and response to school screening and perceptions of school leaders on the ease of connecting youth with mental health care.

## Methods

The RAND American School Leader Panel (ASLP) is a nationally representative sample of kindergarten through grade 12 public school principals. School-level data from the National Center for Education Statistics Common Core of Data were linked to October 2024 survey responses to explore differences by grade level, school size, racial and ethnic composition, urbanicity, census region, and neighborhood poverty. The school’s county was mapped to the Health Resources and Services Administration’s mental health professional shortage areas. ASLP details are available elsewhere.^4^

Adapting the US Centers for Disease Control and Prevention School Health Policies and Practices Study questions on mental health screening,^3^ we asked 3 questions. First, does the school district mandate screening students for mental health problems? Second, what steps are taken if a student is identified as having anxiety or depression, 2 commonly identified mental health diagnoses with established screening recommendations and tools? Third, how easy or difficult is it to ensure students identified with anxiety or depression receive appropriate mental health services? The questions are in Supplement 1.

We report unadjusted frequencies for the questions, then report the results of Wald tests completed after bivariate logistic regression analyses examining school screening and school or county characteristics. All analyses utilized ASLP survey weights and were conducted using Stata version 18.0 (StataCorp). A 2-sided P<.05 was used for statistical significance. The Harvard Pilgrim Health Care Institute institutional review board deemed the research exempt from review because the study is a secondary analysis of deidentified data. The study follows the STROBE reporting guideline for cross-sectional studies.

## Results

Of the 1019 school principals responding, the denominator varies depending on the question: 30.5% reported their district mandating schools screen students for mental health problems (Figure). If a student is identified as having anxiety or depression, most principals reported their school typically notifies the student’s parents (weighted percentage, 79.3%), offers in-person treatment (weighted percentage, 72.3%), and refers the student to a community mental health care professional (weighted percentage, 53.0%). In terms of ease, 40.9% of principals reported ensuring student receipt of appropriate care was very hard or somewhat hard, compared with 38.1% who reported it was very easy or somewhat easy. Regression analyses indicated higher rates of screening in school sizes of 450 or more students (odds ratio [OR].1.32; 95% CI, 1.00-1.75; *P* = .048) and in districts with mostly racial and ethnic minority groups as the student population (OR, 1.48; 95% CI, 1.12-1.96; *P* = .01) (Table). No other differences were statistically significant.

**Figure.**
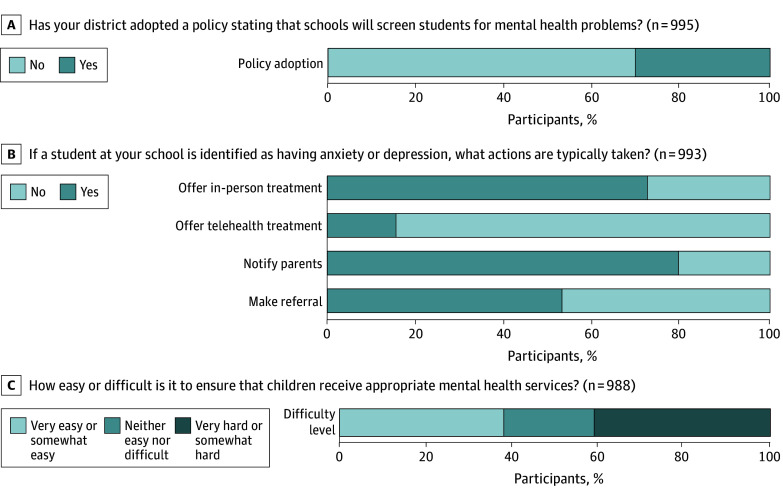
School Screening Policies and Difficulty of Receiving Appropriate Care Survey respondents are public school principals in the American School Leader Panel. All percentage values reported use survey weights and responses are restricted to nonmissing.

**Table.** Characteristics of Schools That Have a Screening Policy^a^

Characteristic^b^	Frequency, No. (%)^c^		Univariate logistic regression	
	Yes (n=302)	No (n=693)	Odds ratio (95% CI)^d^	Wald test *P* value^e^
Neighborhood poverty				
High neighborhood poverty (income-to-poverty ratio ≤200)	60 (30.9)	136 (69.1)	1 [Reference]	.65
Middle neighborhood poverty (income-to-poverty ratio 201-400)	163 (29.3)	392 (70.7)	0.93 (0.65-1.33)	
Low neighborhood poverty (income-to-poverty ratio ≥401)	73 (32.8)	152 (67.2)	1.09 (0.71-1.66)	
Grade level				
Elementary	180 (31.5)	391 (68.5)	1 [Reference]	.55
Middle	56 (31.7)	125 (68.3)	1.01 (0.69-1.46)	
High	58 (27.5)	153 (72.5)	0.82 (0.58-1.18)	
School size, No. of students				
<449	145 (27.7)	380 (72.3)	1 [Reference]	.048
≥450	154 (33.7)	305 (66.3)	1.32 (1.00-1.75)	
Race and ethnicity of student population^f^				
Most students with White race	146 (26.5)	393 (73.5)	1 [Reference]	.01
Most students with other race and ethnicity	153 (34.9)	292 (65.1)	1.48 (1.12-1.96)	
Urbanicity				
Urban	85 (32.2)	182 (67.8)	1 [Reference]	.10
Suburban	109 (34.0)	212 (66.1)	1.08 (0.76-1.54)	
Town/rural	106 (26.8)	296 (73.2)	0.77 (0.54-1.09)	
Census region				
Midwest	80 (26.9)	222 (73.1)	1 [Reference]	.41
Northeast	52 (30.1)	117 (69.9)	1.17 (0.77-1.79)	
South	104 (32.9)	211 (67.1)	1.33 (0.94-1.90)	
West	65 (32.2)	140 (67.8)	1.29 (0.87-1.93)	
County mental health professional shortage area^g^				
None of the county designated as a shortage area	16 (25.1)	46 (74.9)	1 [Reference]	.26
The whole county was designated as a shortage area	65 (27.4)	174 (72.6)	1.12 (0.59-2.15)	
≥1 Parts of the county was designated as a shortage area	215 (32.2)	459 (67.8)	1.41 (0.77-2.59)	

^a^ Survey respondents are principals in the American School Leader Panel responding to the question, Has your district adopted a policy stating that schools will screen students for mental health problems?

^b^ School characteristics are from the National Center for Education Statistics’ Common Core of Data.

^c^ All percentages reported use survey weights and are row percentages. Frequency No.’s are unweighted.

^d^ The odds ratio and 95% CI come from a univariate regression assessing whether the school has a screening policy or not.

^e^ *P* values are from a Wald test completed after a univariate logistic regressions between the characteristic and whether the school has a screening policy or not.

^f^ In the source data file, the specific terms used were “majority White” and “majority students of color” for convenience without further explanation available.

^g^ The county mental health professional shortage is from the Health Resources & Service Administration.

## Discussion

Results of this study suggest that there are multiple barriers to mental health screening in schools including a lack of resources and knowledge of screening mechanics and concerns of increased workload after identifying students.^5^ Policies that promote federal and state funding for school mental health services, reimbursement for school-based mental health screening, and adequate school mental health staffing ratios may increase screening rates and successful connection to care.^6^

This study has several limitations. Results are limited to a nationally representative cross-sectional sample of public schools and may not generalize to private schools. Additionally, we do not have information on school-based early intervention for subthreshold mental health symptoms or health care utilization or quality. Nonetheless, our study provides timely information regarding current school mental health screening and referral efforts nationally.

## Notes

Supplement 1. **eMethods.** RAND American Educator Panels: Fall 2024 Omnibus Principal Survey

Supplement 2.
Data Sharing Statement

## References

[1] Office of the Surgeon General. Protecting Youth Mental Health: The U.S. Surgeon General’s Advisory. US Department of Health and Human Services; 2021. Accessed March 23, 2024. https://www.ncbi.nlm.nih.gov/books/NBK575984/

[2] Foster S, Rollefson M, Doksum T, Noonan D, Robinson G, Teich J. School Mental Health Services in the United States, 2002-2003. SAMHSA’s National Clearinghouse for Alcohol and Drug Information (NCADI); 2005. Accessed May 13, 2025. https://eric.ed.gov/?id=ED499056

[3] McCabe EM, Jameson BE, Strauss SM. Mental Health Screenings: Practices and Patterns of These and Other Health Screenings in U.S. School Districts. J Sch Nurs. 2024;40(2):144-154. doi:10.1177/10598405211056647 34796761

[4] Robbins MW, Grant D. RAND American Educator Panels Technical Description. RAND Corporation; 2020. Accessed February 2, 2025. https://www.rand.org/pubs/research_reports/RR3104.html

[5] Burns JR, Rapee RM. From barriers to implementation: Advancing universal mental health screening in schools. J Psychol Couns Sch. 2021;31(2):172-183. doi:10.1017/jgc.2021.17

[6] National Center for Education Statistics. School Pulse Panel - Interactive Results. Accessed February 18, 2025. https://nces.ed.gov/surveys/spp/results.asp#mentalhealth-march24-chart5

