# Supplementary material for: Screening for Mental Health Problems in US Public Schools
Source: JAMA Netw Open. 2025 Jul 18;8(7):e2521896. doi: 10.1001/jamanetworkopen.2025.21896 (PMC12274975; doi:10.1001/jamanetworkopen.2025.21896)
Supplement: Supplement 1. — eMethods. RAND American Educator Panels: Fall 2024 Omnibus Principal Survey [file jamanetwopen-e2521896-s001.pdf]

Supplemental Online Content

Cantor J, McBain RK, Rankine J, et al. Screening for mental health problems within schools in the US. *JAMA Netw Open.* 2025;8(7):e2521896. doi:10.1001/jamanetworkopen.2025.21896

eMethods. RAND American Educator Panels: Fall 2024 Omnibus Principal Survey

This supplemental material has been provided by the authors to give readers additional information about their work.

© 2025 Cantor J et al. *JAMA Network Open*.

RAND American Educator Panels: Fall 2024 Omnibus Principal Survey

Has your district adopted a policy stating that schools will screen students for mental health problems?
SELECT ONE RESPONSE
1 Yes
0 No

If a student at your school is identified as having anxiety or depression through screening or otherwise, what actions are typically taken?
SELECT ALL THAT APPLY
01 We offer in-person treatment by a counselor/social worker/nurse employed by the school or school district.
02 We offer treatment through telemedicine (aka telehealth) with a counselor/social worker/nurse employed by the school or school district.
03 We offer in-person treatment through a provider that contracts with the school or school district.
04 We offer treatment through telemedicine (aka telehealth) through a provider that contracts with the school or school district.
05 We notify the student's parents.
06 We refer the student to a community healthcare provider.
91 Other (please describe:)
99 None of the above.

© 2025 Cantor J et al. *JAMA Network Open*.

Considering all the available options at your school or school district, how easy or difficult is it to ensure that children identified as having anxiety or depression at your school receive appropriate mental health services?

SELECT ONE RESPONSE

01 Very easy
02 Somewhat easy
03 Neither easy nor difficult
04 Somewhat difficult
05 Very difficult

© 2025 Cantor J et al. *JAMA Network Open*.
