# Supplementary material for: Screening for Mental Health Problems in US Public Schools
Source: JAMA Netw Open. 2025 Jul 18;8(7):e2521896. doi: 10.1001/jamanetworkopen.2025.21896 (PMC12274975; doi:10.1001/jamanetworkopen.2025.21896)
Supplement: Supplement 2. — Data Sharing Statement [file jamanetwopen-e2521896-s002.pdf]

Data Sharing Statement

Cantor. Screening for Mental Health Problems in US Public Schools. *JAMA Netw Open*. Published July 18, 2025. doi:10.1001/jamanetworkopen.2025.21896

Data
Data available: No

Additional Information
Explanation for why data not available: Requires the signing of a data use agreement.
